# Expanding Lung Volume Reduction Surgery Indications: Outcomes in Patients Beyond Conventional National Emphysema Treatment Trial Criteria

**DOI:** 10.1093/icvts/ivaf274

**Published:** 2025-11-18

**Authors:** Christelle M Vandervelde, Anthony Meyers, Anaïs David, Sofian Bouneb, Stephanie Everaerts, Wim Janssens, Walter Weder, Laurens J Ceulemans

**Affiliations:** Department of Thoracic Surgery, University Hospitals Leuven, Leuven, Belgium; Department of Chronic Diseases and Metabolism Laboratory of Pneumology and Thoracic Surgery (BREATHE), KU Leuven, Leuven, Belgium; Department of Thoracic Surgery, University Hospitals Leuven, Leuven, Belgium; Department of Thoracic Surgery, University Hospitals Leuven, Leuven, Belgium; Department of Anaesthesiology, University Hospitals Leuven, Leuven, Belgium; Department of Cardiovascular Sciences, Division of Anaesthesiology and Algology, KU Leuven, Leuven, Belgium; Department of Chronic Diseases and Metabolism Laboratory of Pneumology and Thoracic Surgery (BREATHE), KU Leuven, Leuven, Belgium; Department of Respiratory Diseases, University Hospitals Leuven, Leuven, Belgium; Department of Chronic Diseases and Metabolism Laboratory of Pneumology and Thoracic Surgery (BREATHE), KU Leuven, Leuven, Belgium; Department of Respiratory Diseases, University Hospitals Leuven, Leuven, Belgium; Department of Thoracic Surgery, Klinik Bethanien, Zurich, Switzerland; Department of Thoracic Surgery, University Hospitals Leuven, Leuven, Belgium; Department of Chronic Diseases and Metabolism Laboratory of Pneumology and Thoracic Surgery (BREATHE), KU Leuven, Leuven, Belgium

**Keywords:** COPD, emphysema, lung volume reduction surgery, selection criteria

## Abstract

**Objectives:**

Lung volume reduction surgery (LVRS) is guided by strict selection criteria from the National Emphysema Treatment Trial (NETT) to minimize risk and optimize outcomes. However, emerging evidence suggests that rigid cutoffs may exclude patients who could benefit. This study aimed to identify beyond-criteria patients undergoing LVRS and compare their outcomes with standard-criteria patients.

**Methods:**

This single-centre retrospective analysis of a prospectively maintained database included all LVRS procedures from August 2019 until November 2024. Patients were classified as beyond-criteria if they met two or more of the following: age ≥ 75 years, body mass index (BMI) < 18.5 kg/m^2^, forced expiratory volume in 1 second (FEV_1_) < 20%pred, diffusing capacity for carbon monoxide (DLCO) < 20%pred, 6-minute walk distance (6MWD) < 140 m, homogeneous emphysema, systolic pulmonary arterial pressure (sPAP) > 35 mmHg, or prior thoracic interventions. Complications (Clavien-Dindo) and functional outcomes were assessed at 3, 6, and 12 months.

**Results:**

Twenty-one procedures were performed in 18 beyond-criteria patients versus 227 procedures in 191 standard-criteria patients. Among beyond-criteria patients: age ≥ 75 years (*n* = 3), BMI < 18.5 kg/m^2^ (*n* = 10), FEV_1_ < 20%pred (*n* = 5), DLCO < 20%pred (*n* = 1), 6MWD < 140 m (*n* = 1), homogeneous emphysema (*n* = 3), sPAP > 35 mmHg (*n* = 12), and prior thoracic intervention (*n* = 7). Complication rates were comparable (38% vs. 42%, *P *= .819 [95% CI, 0.93-1.10]), as were prolonged air leaks and hospital stay. One 30-day LVRS-related death (0.4%) occurred in the standard group. Functional and quality of life measures improved in both groups.

**Conclusions:**

Beyond-criteria patients can be considered for LVRS when guided by careful multidisciplinary evaluation, with meaningful improvement in experienced centers.

## Graphical abstract

**Figure:**
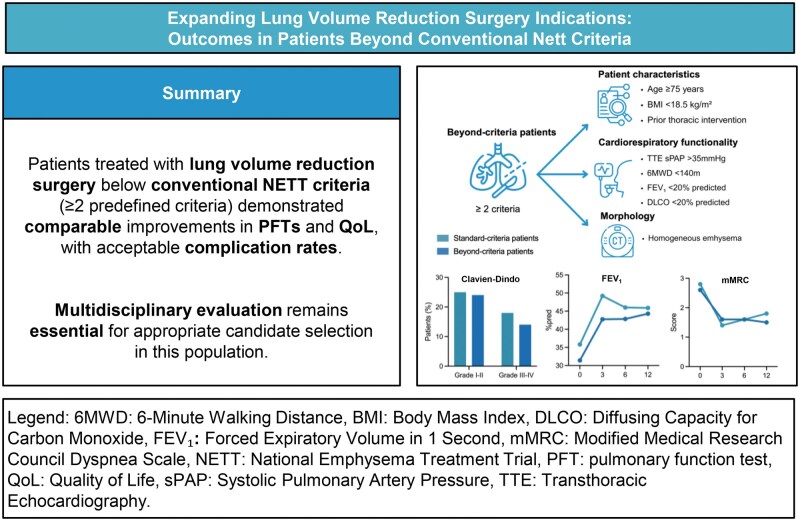

## INTRODUCTION

Lung volume reduction surgery (LVRS) is a proven treatment for severe emphysema, a subtype of chronic obstructive pulmonary disease (COPD) marked by alveolar destruction resulting in air trapping and impaired gas exchange, leading to respiratory insufficiency and dyspnea.^1^^,^^2^ Resecting the most damaged, non-functioning lung tissue, reduces hyperinflation and restores elastic recoil, leading to improved lung function and exercise capacity.^3^

The selection criteria for LVRS were formally established by the National Emphysema Treatment Trial (NETT), which compared maximal medical therapy to LVRS.^4^ It was demonstrated that LVRS improved quality of life (QoL) and functional capacity in properly selected patients. However, a pre-trial analysis identified a high-risk subgroup (forced expiratory volume in 1 second (FEV_1_) < 20%pred or diffusing capacity for carbon monoxide (DLCO) < 20% pred) with a homogeneous morphology.^5^ This resulted in favouring patients with upper-lobe predominant heterogeneous emphysema and low baseline exercise capacity, while discouraging surgery in patients with homogeneous emphysema and severely impaired lung function.

Despite these criteria, many centers continued offering LVRS in carefully selected subgroups of emphysema patients beyond the traditional NETT criteria. These clinical observations provided evidence that patients with previously considered contraindications, such as homogeneous emphysema,^6^ pulmonary hypertension (> 35 mmHg),^7^ advanced age (≥ 75 years),^8^ and DLCO less than 20%pred,^9^ achieved meaningful improvements. These findings support reconsidering rigid adherence to NETT criteria in favor of a more individualized selection strategy.^10^

To further investigate the role of LVRS in this broader patient population, this study evaluated outcomes in individuals who would traditionally be excluded. Building on prior work demonstrating that implementation of an enhanced recovery protocol (ERP) for LVRS can reduce surgical morbidity,^11^ it was hypothesized that, under optimized perioperative care, carefully selected beyond-criteria patients could still achieve meaningful functional improvement and clinical benefit from LVRS.

This study aims to define the beyond-criteria cohort and descriptively compare their outcomes with those of patients meeting traditional NETT criteria.

## METHODS

### Ethical statement

The research ethics committee of UZ/KU Leuven approved the long-term follow-up study on 22 January 2021 (reference number: S64530). Prospective data collection was conducted following written informed consent from all participants.

### Study population and design

**Figure 1.**
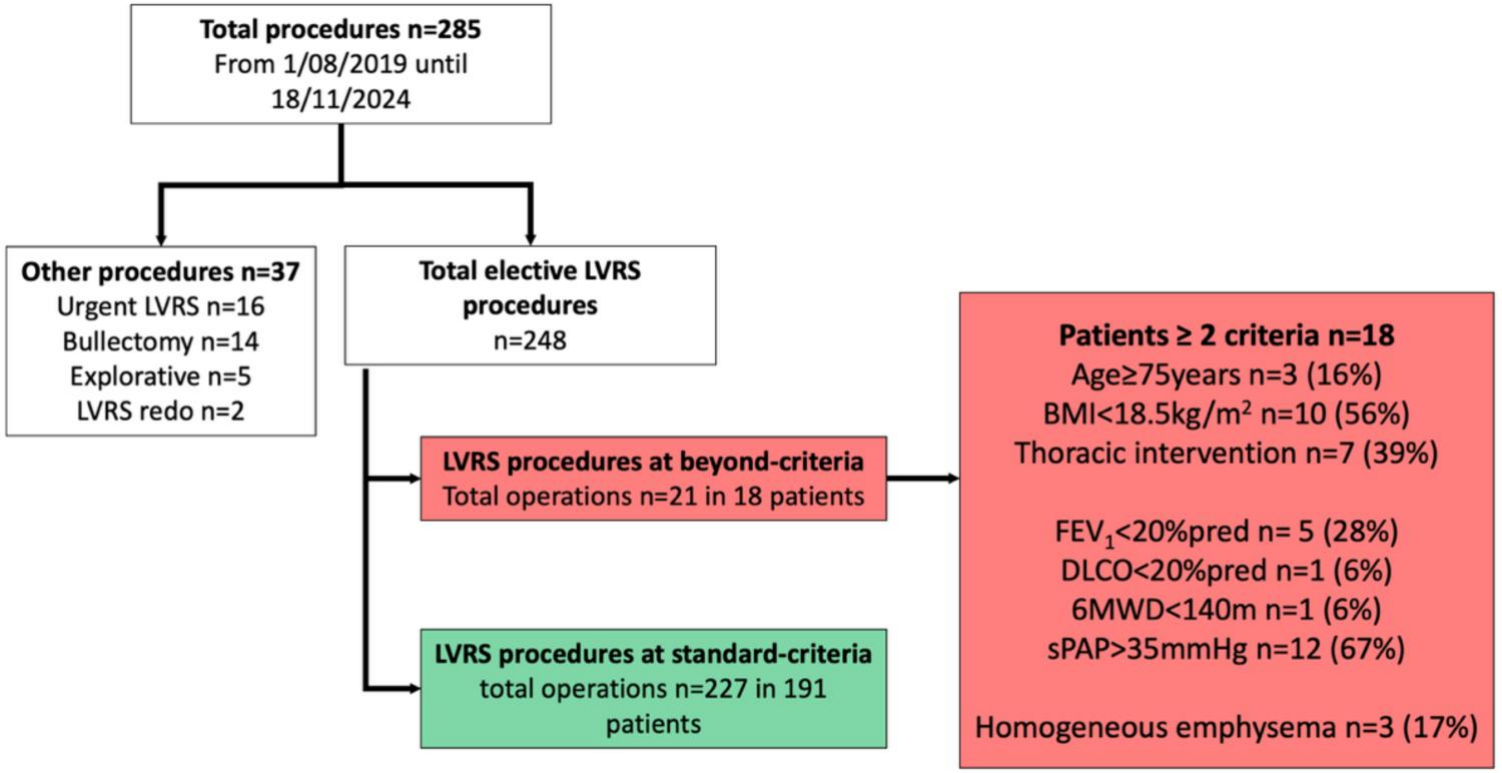
Flowchart of the Study Cohort. All lung volume reduction surgery (LVRS) procedures performed between August 1, 2019, and November 18, 2024. Of the 285 procedures, 248 were elective and included in the analysis. Patients were classified as either standard-criteria (*n* = 227 in 191 patients) or beyond-criteria (*n* = 21 in 18 patients), based on presence of 2 or more predefined criteria. These included age ≥ 75 years, BMI < 18.5 kg/m^2^, FEV_1_ < 20%predicted, DLCO < 20%predicted, 6MWD < 140 m, homogeneous emphysema, sPAP > 35 mmHg, or prior thoracic interventions. 6MWD, 6-minute walk distance; BMI, body mass index; DLCO, diffusing capacity for carbon monoxide; FEV_1_, forced expiratory volume in 1 second; LVRS, lung volume reduction surgery; sPAP, systolic pulmonary artery pressure

All consecutive patients who underwent LVRS at University Hospitals Leuven between August 2019 and November 2024 were identified. Urgent procedures (*n* = 16), bullectomy (*n* = 14), exploratory cases (*n* = 5), or redo LVRS (*n* = 2) were excluded, resulting in a total of 248 included procedures (**Figure 1**). All patients underwent video-assisted thoracoscopic surgery (VATS), performed unilaterally or bilaterally, without conversion to open surgery.

### Assessment eligibility

Eligibility was determined case-by-case by a multidisciplinary team consisting of thoracic surgeons, pulmonologists, radiologists, anaesthesiologists and physiotherapists.^11^ Standardized assessments included 6-minute walk distance (6MWD) and pulmonary function testing (PFT), including spirometry, body plethysmography, and diffusing capacity (Vyaire Medical) per ATS/ERS guidelines. Morphology was classified per Weder et al^12^ by high-resolution computed tomography (HRCT) in homogeneous, markedly heterogeneous, and intermediate heterogeneous distributed emphysema. Pulmonary arterial hypertension (PAH) was routinely screened using transthoracic echocardiography (TTE), with systolic pulmonary arterial pressure (sPAP) < 35 mmHg considered normal (or reported as normal if no numerical value was provided); right heart catheterization (RHC) was used to rule out pulmonary hypertension when TTE findings were inconclusive or suggestive for elevated pressures, and was adopted as a routine assessment later in the study period. Potential risk factors (e.g., elevated CO_2_, low 6MWD, etc.) were assessed individually, allowing inclusion of beyond-criteria patients.^13^ No predefined optimization protocols were applied for beyond-criteria cases.

### Beyond-criteria patients

Patients were assigned to the beyond-criteria group if they met ≥ two of the following criteria, predefined across three domains. Patient characteristics included age ≥ 75 years, body mass index (BMI) < 18.5 kg/m^2^, and previous thoracic interventions (bullectomy, wedge excision, pleurodesis, radiotherapy, or chest tube-treated pneumothorax). Cardiorespiratory functionality criteria included FEV_1_ < 20%pred, DLCO < 20%pred, 6MWD < 140 m, and sPAP > 35 mmHg (TTE). Morphologic criteria included homogeneous emphysema (**Table 1**). Patients not meeting at least two criteria were assigned to the standard-criteria group.

**Table 1.** Patient Demographics and Clinical Data

	Standard-criteria	Beyond-criteria	P
**Patient characteristics**			
Patients, n	191	18	
Operations, n	227	21	
Age, years	63.0 (43.0-78.0) *n* = 191	65.0 (50.0-77.0) *n* = 18	.270
Female, n (%)	87 (46)	12 (67)	.137
BMI (kg/m^2^)	23.8 (14.5-31.9) *n* = 191	18.0 (13.9-29.2) *n* = 18	**<.000**
Pack years	40.0 (0.0-141.0) *n* = 186	40.0 (15.0-50.0) *n* = 18	.427
**Medical history**			
Arterial hypertension, n (%)	67 (35)	4 (22)	.311
Diabetes mellitus, n (%)	11 (6)	2 (11)	.310
Hypercholesterolemia, n (%)	68 (36)	7 (39)	.801
Cardiac arrhythmias, n (%)	11 (6)	0	.604
Coronary disease, n (%)	31 (16)	3 (17)	>.999
Asthma, n (%)	32 (16)	2 (11)	.746
Pneumothorax, n (%)	13 (7)	5 (28)	**.011**
Pleurodesis, n (%)	3 (2)	1 (6)	.318
Bullectomy, n (%)	2 (1)	1 (6)	.238
Wedge resection, n (%)	0	1 (5)	.086
Endobronchial valve, n (%)	18 (9)	4 (28)	**.033**
Exacerbations past year, n (%)	65 (34)	6 (33)	>.999
**Therapy**			
Chronic systemic steroids, n (%)	33 (17)	3 (17)	>.999
Azithromycin, n (%)	83 (43)	5 (28)	.223
Oxygen, n (%)	73 (38)	10 (56)	.207
PaCO_2_ (mmHg)	39.6 (29.0-60.0) *n* = 24	39.9 (37.3-40.4) *n* = 3	.821
**Quality of life**			
CAT	22.0 (6.0-39.0) *n* = 148	20.0 (12.0-29.0) *n* = 15	.477
mMRC	3.0 (0.0-4.0) *n* = 172	3.0 (2.0-4.0) *n* = 16	.178
SGRQ	62.0 (26.8-90.0) *n* = 130	55.5 (28.0-76.0) *n* = 11	.517
**Cardiorespiratory functionality**			
FEV_1_ (L)	0.9 (0.4-2.5) *n* = 190	0.7 (0.4-2.1) *n* = 18	
%pred	31.0 (18.0-66.0) *n* = 191	30.0 (13.0-58.0) *n* = 18	.308
RV (L)	4.8 (2.0-8.5) *n* = 183	5.1 (3.1-8.4) *n* = 17	
%pred	222.5 (90.0-401.0) *n* = 190	231.0 (178.0-392.0) *n* = 18	.626
DLCO (%pred)	38.0 (20.0-71.0) *n* = 185	29.5 (18.0-45.0) *n* = 16	**.001**
6MWD (m)	367.5 (106.0-608.0) *n* = 180	332.0 (120.0-577.0) *n* = 17	.655
sPAP (transthoracic echocardiography)	30.0 (12.0-68.0) *n* = 96	39.0 (18.0-61.0) *n* = 15	**.000**
sPAP (right heart catheterization)	37.0 (21.0-50.0) *n* = 21	39.0 (29.0-45.0) *n* = 7	.541
**Morphology**			
Homogeneous, n (%)	6 (3)	3 (17)	**.033**
Intermediate heterogeneous, n (%)	108 (57)	9 (50)	.626
Heterogeneous, n (%)	76 (40)	6 (33)	.801

Patient demographics and clinical data in standard versus beyond-criteria patients. Data are presented as median (range) or number (%). Categorical data: Fisher’s exact test. Continuous variables: Mann-Whitney tests. 6MWD, the 6-minute walk distance; BMI, body mass index; CAT, COPD Assessment Test; DLCO, diffusing capacity of the lung for carbon monoxide; FEV_1_, forced expiratory volume in 1 second; FVC, forced vital capacity; mMRC, Modified Medical Research Council dyspnoea scale; RV, residual volume; SGRQ, St George’s Respiratory Questionnaire; sPAP, systolic pulmonary artery pressure. Values in bold are statistically significant.

**Table 2.** Surgical Outcomes

Clavien-Dindo classification	Standard-criteria, n = 227	Beyond-criteria, n = 21	P
Patients with at least 1 complication, n (%)	97 (42)	8 (38)	.819
**Minor (grade 1-2)**	57 (25)	5 (24)	>.999
Prolonged air leak (>7 days)	38 (17)	2 (10)	.543
Subcutaneous emphysema	36 (16)	2 (10)	
Urinary tract infection	3 (1)	1 (5)	
Atrial fibrillation	12 (5)	0	
Pneumonia	8 (4)	0	
Urinary retention	13 (8)	1 (5)	
Heimlich valve	5 (2)	1 (5)	
Miscellaneous^a^	7 (3)	1 (5)	
**Major (grade 3-4)**	40 (18)	3 (14)	>.999
VAC for subcutaneous emphysema	8 (4)	1 (5)	
Chest tube re-insertion	6 (3)	2 (10)	
Reintervention for prolonged air leak	18 (8)	0	
Respiratory failure	2 (1)	0	
Acute kidney injury	6 (3)	0	
Miscellaneous^b^	7 (3)	0	
**30-day LVRS-related mortality (grade 5**)	1^c^ (0.4)	0	>.999
**90-day mortality**	3 (1)	0	>.999
**Surgical outcomes**			
Length of stay (days)	7.0 (2.0-73.0) *n* = 226	5.0 (2.0-12.0) *n* = 21	.370
ICU admission	7 (3)	1 (5)	.513
Air leak POD1	64 (28)	4 (19)	.452
Chest tube duration (days)	4.0 (1.0-74.0) *n* = 226	3.0 (2.0-15.0) *n* = 21	.172
Readmission first 30 days	16 (7)	1 (5)	>.999
Exacerbation first 30 days	18 (8)	0	.378

Postoperative outcomes (Clavien-Dindo) for procedures in standard versus beyond-criteria patients. Data are shown as median (range) or number (%). Categorical data: Fisher’s exact test. Continuous variables: Mann-Whitney tests. ICU, intensive care unit; LVRS, lung volume reduction surgery; POD1, postoperative day 1; VAC: vacuum-assisted closure.

a COVID-19 infection, pulmonary oedema.

b Tension pneumothorax, laparotomy for small bowl obstruction.

c Cause of death: acute on chronic kidney failure.

### Surgical and functional outcomes

Primary outcome was postoperative complications (Clavien-Dindo),^14^ other surgical outcomes included chest-tube duration (days), prolonged air leak (PAL) (> 7 days), length of stay (LOS) (days), in-hospital, 30- and 90-day mortality.

Functional outcomes were assessed at baseline and 3, 6, and 12 months postoperative. PFTs included FEV_1_, residual volume (RV), and DLCO. Exercise capacity was assessed by 6MWD and QoL using modified Medical Research Council (mMRC) dyspnea scale, COPD Assessment Test (CAT), and St. George’s Respiratory Questionnaire (SGRQ).

### Statistical analysis

Data were presented as median (range, minimum-maximum). Categorical variables were compared using Fisher’s exact, and continuous variables using Mann-Whitney U. For key outcomes, effects estimates were reported with 95% confidence intervals [95% CI]. Analyses were based on available cases without imputation. All *P*-values were two-tailed and unadjusted for multiplicity. A value of *P* < 0.05 was considered statistically significant. Analyses were performed using GraphPad Prism (v.10.4.1; macOS).

## RESULTS

### Study population

A total of 248 elective LVRS procedures were performed, including 21 in 18 beyond-criteria patients and 227 in 191 standard-criteria patients (**Figure 1**).

### Beyond-criteria patients

**Figure 2.**
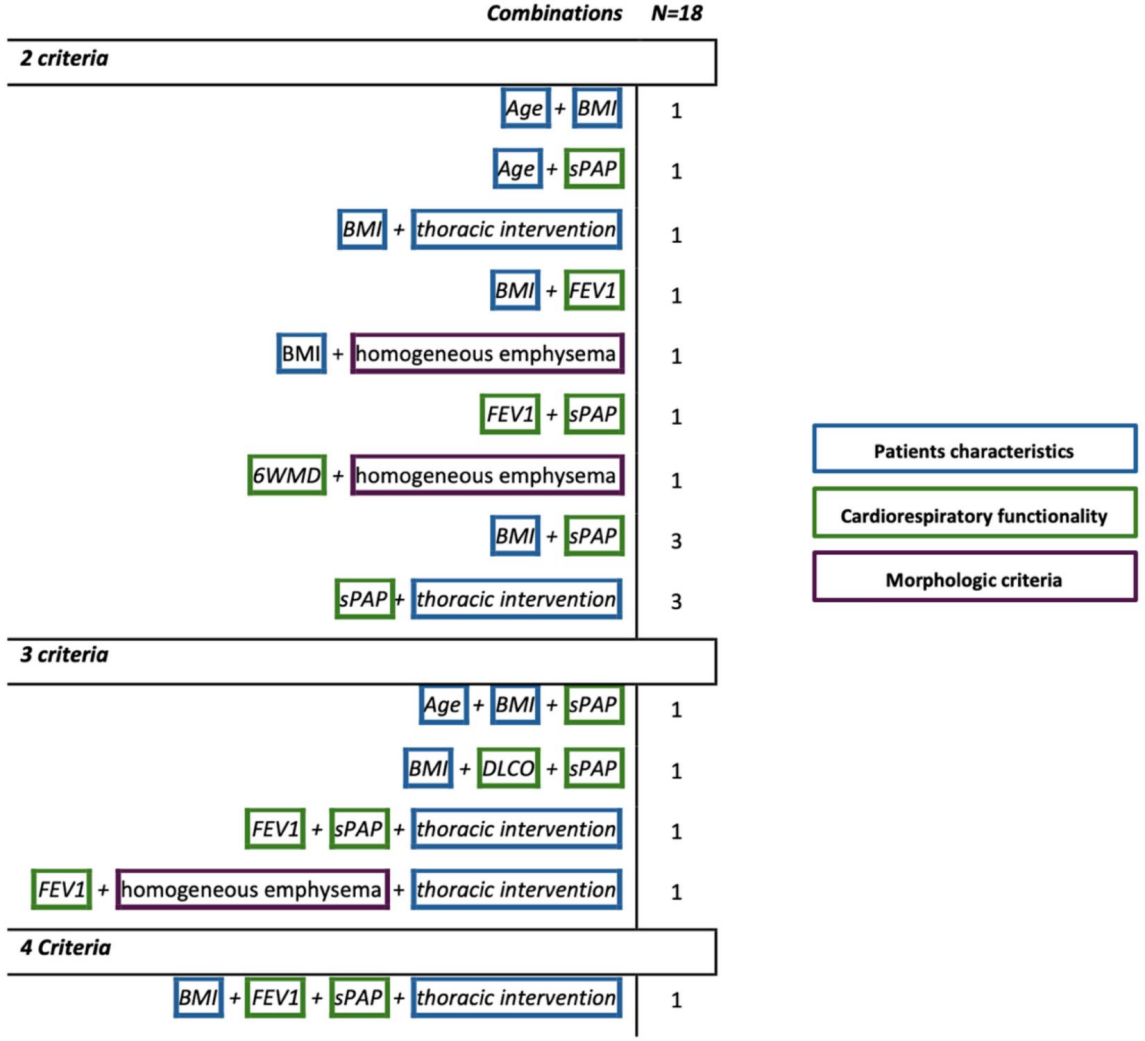
Beyond-Criteria Combinations. Overview of clinical combination characteristics in beyond-criteria patients (*n*=18), grouped into 3 categories: patient characteristics, cardiorespiratory function, and morphology. Body mass index (BMI), diffusing capacity of the lung for carbon monoxide (DLCO), forced expiratory volume in 1 second (FEV_1_), 6-minute walk distance (6MWD), systolic pulmonary artery pressure (sPAP), and previous thoracic intervention

Most frequent risk factors in beyond-criteria patients were TTE sPAP > 35 mmHg *n* = 12 (67%), BMI < 18.5 kg/m^2^  *n* = 10 (56%), and prior thoracic interventions *n* = 7 (39%), followed by FEV_1 _< 20%pred *n* = 5 (28%) and homogeneous emphysema *n* = 3 (17%) (**Figure 1**). Age ≥ 75 years, DLCO < 20%pred, and 6MWD < 140 m were observed in 3 (16%), 1 (6%), and 1 (6%) patient(s), respectively (**Figures 1** and **2**; **Figure S1**).

### Beyond- versus standard-criteria patients

#### Baseline characteristics

Regarding patient characteristics, the beyond-criteria group had a significantly lower BMI compared to the standard-criteria group (18.0 vs. 23.8 kg/m^2^, *P *< .001 [95% CI, 2.56-6.38]). History of pneumothorax (28% vs. 7%, *P *< .011 [95% CI, 0.53-0.94]) and previous endobronchial valve treatment (28% vs. 9%, *P *= .033 [95% CI, 0.62-0.98]) was more frequent in the beyond-criteria cohort (**Table 1**).

Concerning the cardiorespiratory functionality criteria, baseline DLCO was significantly lower in beyond-criteria patients (30%pred vs. 38%pred, *P *= .001 [95% CI, 3.00-13.00]). FEV_1_ (30%pred vs. 31%pred, *P *= .308 [95% CI, -3.00 to 9.00]), RV (231%pred vs. 223%pred, *P *= .626 [95% CI, -26.00 to 15.00]), CAT (20 vs. 22, *P *= .477 [95% CI, -2.00 to 4.00]), mMRC (3 in both, *P *= .178 [95% CI, -1.00 to 0.00]), SGRQ-scores (55.5 vs. 62.0, *P *= .517 [95% CI, -6.40 to 12.80]), and 6MWD (332 m vs. 368 m, *P *= .655 [95% CI, -46.00 to 67.00]) were comparable between groups. TTE sPAP was higher in beyond-criteria (39.0 vs. 30.0 mmHg, *P *= .001 [95% CI, -18.00 to -6.00]), RHC sPAP did not differ (*P *= .541 [95% CI, -10.00 to 4.00]).

In terms of morphology, homogeneous emphysema was more frequent in the beyond-criteria group (17% vs. 3%, *P *= .032 [95% CI, 0.38-0.96]).

#### Surgical outcome

Unilateral LVRS was more frequent in the beyond-criteria (43% vs. 31%, *P *= .330 [95% CI, 0.86-1.03]), while one-stage bilateral procedures were more common in the standard-criteria group (29% vs. 36%, *P *= .635 [95% CI, 0.94-1.11]). Staged bilateral procedures were comparable (14% vs. 16%, *P *> .999 [95% CI, 0.87-1.09]). Surgery duration was similar (**Table S2**).

Eight patients (38%) in the beyond-criteria and 97 (42%) in the standard-criteria cohort developed postoperative complications (*P *= .819 [95% CI, 0.93-1.10]). Grade I-II were comparable (24% vs. 25%, *P *> .999 [95% CI, 0.90-1.08]), with PAL being the most common (10% vs. 17%, *P *= .543 [95% CI, 0.92-1.12]). Grade III-IV occurred in 14% and 18% of patients (**Table 2**).

No 30-day mortality occurred in the beyond-criteria, vs. one death (0.4%: acute on chronic kidney failure), in the standard group. There were no reinterventions for PAL, respiratory failure, or acute kidney injury in the beyond-criteria group.

No significant differences were observed for air leak on postoperative day 1 (19% vs. 28%, *P *= .452 [95% CI, 0.94-1.12]), chest tube duration (3 vs. 4 days, *P *= .172 [95% CI, 0.00-2.00]), hospital stay (5 vs. 7 days, *P *= .370 [95% CI, -1.00 to 2.00]), intensive care unit (ICU) admission (5% vs. 3%, *P *= .513 [95% CI, 0.58-1.08]), or 30-day exacerbations (0% vs. 8%, *P *= .378 [95% CI, 0.91-2.21]).

#### Functional outcome

**Figure 3.**
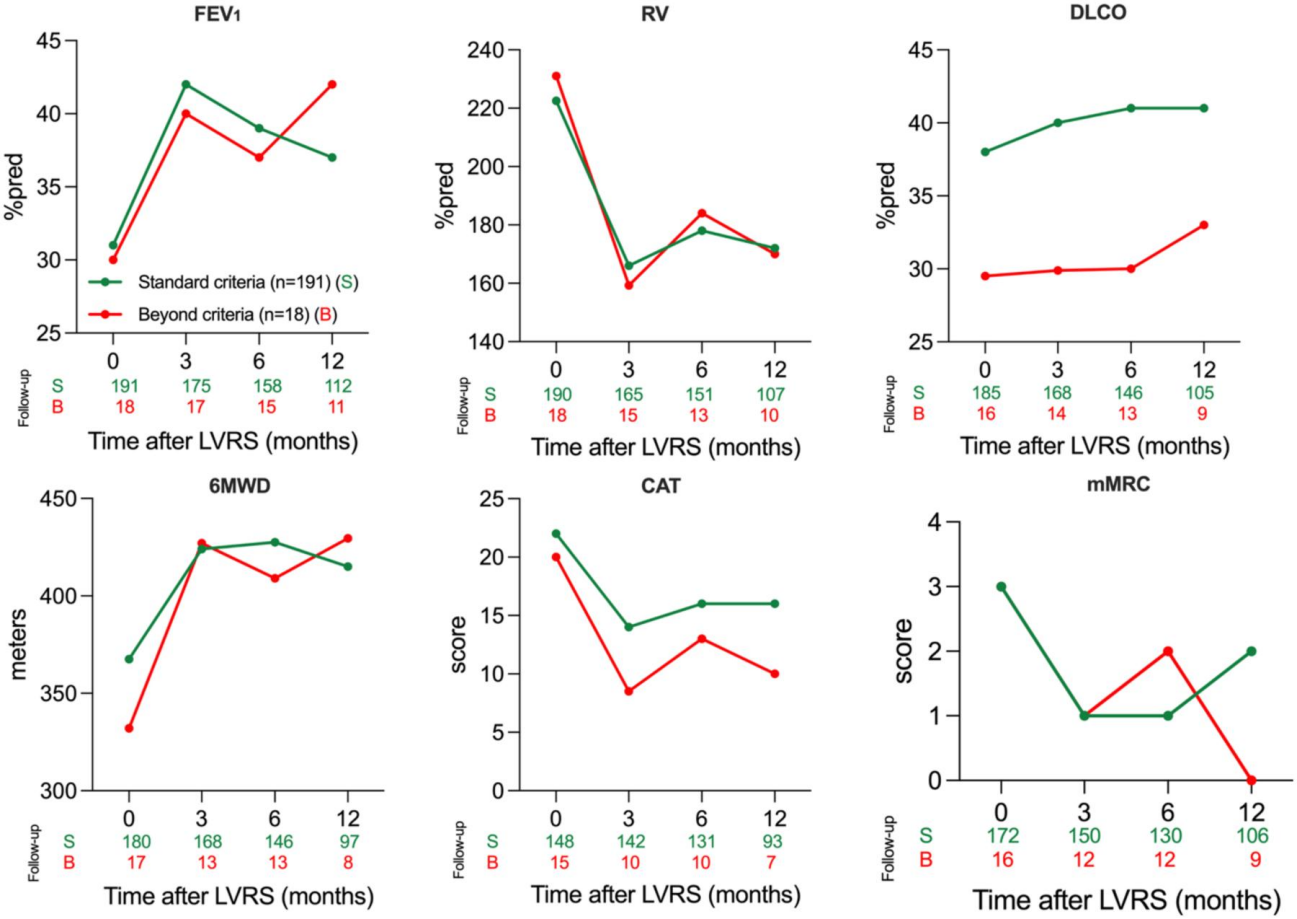
Functional Outcome, Exercise Capacity, and Quality of Life in Standard Versus Beyond-Criteria Patients. Outcomes LVRS for standard versus beyond-criteria patients, measured at baseline, 3, 6, and 12 months postoperatively with indication of patients in follow-up. 6MWD, 6-minute walk distance; BMI, body mass index; CAT, COPD Assessment Test; DLCO, diffusing capacity of the lung for carbon monoxide; FEV_1_, forced expiratory volume in 1 second; LVRS, lung volume reduction surgery; mMRC, Modified Medical Research Council dyspnea scale; RV, residual volume; sPAP, systolic pulmonary artery pressure

There were no significant between-group differences at 3, 6, or 12 months, except for DLCO (**Figure 3**; **Table S3**). Improvement over time was observed in both groups. In the beyond-criteria group, FEV_1_ increased to 42%pred at 12 months (*P *= .038 [95% CI, 1.00-27.00]), mMRC improved to 0 (*P *= .002 [95% CI, -3.00 to -1.00]), CAT decreased to 10 (*P *= .003 [95% CI, -15.00 to -4.00]), and SGRQ to 25 (*P *= .001 [95% CI, -49.60 to -13.00]). RV decreased and DLCO increased, although not statistically significant (**Table S4**). In the standard-criteria group, all parameters (FEV_1_, RV, DLCO, mMRC, CAT, and SGRQ) significantly improved (*P *< .05) (**Table S5**). At 12 months, 6MWD improved to 430 m in the beyond-criteria (*P *= .244 [95% CI, -49.00 to 198.00]) and 415 m in the standard-criteria group (*P *= .002 [95% CI, -76.00 to -17.00]) (**Tables S4 and S5**).

## DISCUSSION

This study demonstrated that, in an experienced center, beyond-criteria patients are eligible for LVRS following multidisciplinary assessment, with limited complications, resulting in improvements in lung function and QoL, and no 30-day mortality.

Historically, the NETT defined strict eligibility criteria for LVRS, identifying subgroups at elevated surgical risk (FEV_1_ < 20%pred or DLCO < 20%pred, with homogeneous emphysema).^5^ Additional exclusion criteria were advanced age, PAH, low exercise capacity, and poor nutritional status (low BMI).^4^ However, evolving evidence suggests that excluding patients solely based on these features may be overly conservative.^7–9^^,^^15^^,^^16^

In our study, “beyond-criteria” status required the presence of at least 2 high-risk features, providing a more accurate identification of high-risk candidates than reliance on any single variable. Consequently, isolated findings, such as DLCO < 20%pred (*n* = 1), FEV_1 _< 20%pred (*n* = 9), or homogeneous emphysema (*n* = 12), were not considered sufficient to categorize a patient as beyond-criteria. This contrasts with previous studies that often defined high-risk patients by a single criterium. Beyond-criteria patients often nearly missed the conventional cut-off values (e.g., lowest DLCO 18%pred), representing a near-miss rather than inclusion of extremely poor candidates.

***Patients characteristics***, defined as advanced age, low BMI, and prior thoracic interventions, have traditionally been viewed as relative contraindications due to presumed increased risk. However, age (≥ 75 years) alone is not necessarily associated with poor outcomes.^8^ In contrast, both low BMI and prior thoracic interventions have been associated with adverse outcomes. Although BMI < 18.5 kg/m^2^ has not been directly evaluated in LVRS, Williams et al identified it as an independent risk factor for pulmonary complications following major lung resection.^17^ Thoracic interventions such as chest tube insertion, pleurodesis, or bullectomy can result in pleural adhesions, increasing intraoperative complexity. In a NETT post-hoc analysis, the presence of adhesions was associated with a higher risk of PAL.^18^ Importantly, BMI alone does not reflect body composition and may not distinguish between low muscle- or fat-mass. In our cohort, patients with prior thoracic interventions or low BMI underwent LVRS without increased morbidity. These factors did not inherently contraindicate surgery and when operability was uncertain, surgical exploration was preformed to assess feasibility. To minimize surgical risk, one-stage bilateral LVRS was avoided in our beyond-criteria patients and a unilateral or staged approach was preferred.

In the context of ***cardiorespiratory function***, parameters like severely reduced DLCO and elevated sPAP, traditionally associated as high-risk, have shown favorable outcomes in selected patients. Caviezel et al. reported in patients with DLCO < 20%pred, no 90-day mortality and significant improvements in lung function and QoL.^9^ Thuppal et al. found that patients with mean PAP > 35 mmHg (RHC) had outcomes comparable to those without pulmonary hypertension, with no increase in perioperative morbidity or mortality,^16^ and Caviezel et al. similarly demonstrated favorable outcomes in patients with sPAP > 35 mmHg (TTE).^7^ Subsequently, LVRS in the context of pulmonary hypertension appears to be feasible in patients with pronounced hyperinflation, as reflected by an elevated RV of 224%pred in a study by Thuppal et al. and a RV of 266%pred in a study by Caviezel et al. In contrast, for patients with FEV_1 _<20%pred or 6MWD < 140 m, supportive data remain limited, and concerns are still largely based on the NETT subgroup analysis.

In this study, seven beyond-criteria patients with suspected pulmonary hypertension on TTE (sPAP > 35 mmHg) underwent confirmatory RHC. In all cases, RHC revealed an overestimation of pressures assessed by TTE. However, all measured mPAPs on RHC remained consistent with pulmonary hypertension defined as mPAP > 20 mmHg (**Table S1**). These findings underline the importance of integrating invasive measurements into preoperative assessment. In our series, sPAP in five patients was only measured by TTE, possibly overestimating the true pulmonary hypertension.

Regarding ***morphological characteristics***, Weder et al. showed that LVRS in patients with homogeneous emphysema and severe hyperinflation was associated with low perioperative mortality and sustained functional improvement.^16^ In our cohort, three beyond-criteria patients had homogeneous disease in combination with other risk factors, including prior thoracic radiotherapy, low BMI, or reduced exercise capacity (**Table S1**). Despite these profiles, all three achieved favorable outcomes. This supports that homogeneous morphology alone should not exclude patients from LVRS.

It is recommended that when selection for LVRS deviates from conventional criteria, this requires careful evaluation of the patient’s risk profile. The baseline evaluation of cardiorespiratory function, did not differ between both groups as the presence of a high-risk criterium (e.g., pulmonary hypertension), was always counterbalanced by another cardiorespiratory criteria (e.g., markedly increased RV or heterogeneous disease). Importantly, none of the beyond-criteria were considered absolute contraindications. However, three criteria form a risky triad (DLCO < 20%pred, homogeneous emphysema, and pulmonary hypertension) and should never be combined in a single patient (**Figure 2**; **Table S1**).

While most studies have focused on lower threshold criteria, emerging evidence also challenges the rigidity of upper limit criteria. Our previous findings showed favourable outcome in patients exceeding traditional selection boundaries,^13^ supporting the view that strict cutoffs may exclude candidates who could benefit from LVRS. These results reinforce the need for individualized, phenotype-based evaluation, over fixed thresholds.

Our study has some limitations; as a single-center analysis, its generalizability may be limited. Additionally, assigning equal weight to all criteria may not fully reflect their differing clinical significance. The relatively small cohort reduced statistical power and some observed differences, although not statistically significant, are clinically relevant. Moreover, the observational design with selective dropout during follow-up introduces the potential for attrition bias, which may have influenced outcome interpretation. Nonetheless, these meaningful improvements in the beyond-criteria subgroup are reassuring.

## CONCLUSION

This study shows that LVRS can be a viable option for beyond-criteria patients when selected through careful multidisciplinary assessment. Although these patients presented with a higher baseline risk profile, those who remained in follow-up experienced limited complications and comparable outcome to standard-criteria patients. These results support a more individualized approach to LVRS, where clinical judgement complements, but does not replace, established selection thresholds.

## Supplementary Material

ivaf274_Supplementary_Data

## Data Availability

All relevant data are available on request to the authors.

## References

[1] Mannino DM , Buist AS. Global burden of COPD: risk factors, prevalence, and future trends. Lancet. 2007;370:765-773. 10.1016/S0140-6736(07)61380-4 17765526

[2] Kahnert K, Jörres RA, Behr J, Welte T. The Diagnosis and Treatment of COPD and Its Comorbidities. *Dtsch Arztebl Int*. 2023;120:434-444. 10.3238/arztebl.m2023.027 PMC10478768 36794439

[3] Shah PL , Herth FJ, van Geffen WH, Deslee G, Slebos DJ. Lung volume reduction for emphysema. Lancet Respir Med. 2017;5:147-156. 10.1016/S2213-2600(16)30221-1 27693408

[4] Fishman A , Martinez F, Naunheim K, et al National Emphysema Treatment Trial Research Group. A randomized trial comparing lung-volume–reduction surgery with medical therapy for severe emphysema. New Engl J Med. 2003;348:2059-2073. http://www.nejm.org/doi/abs/10.1056/NEJMoa030287 12759479 10.1056/NEJMoa030287

[5] Fishman A , Fessler H, Martinez F, et al Patients at high risk of death after lung-volume–reduction surgery. N Engl J Med. 2001;345:1075-1083. http://www.nejm.org/doi/abs/10.1056/NEJMoa11798 11596586 10.1056/NEJMoa11798

[6] Weder W , Ceulemans LJ, Opitz I, Schneiter D, Caviezel C. Lung volume reduction surgery in patients with homogeneous emphysema. Thorac Surg Clin. 2021;31:203-209. 10.1016/j.thorsurg.2021.02.007 33926673

[7] Caviezel C , Aruldas C, Franzen D, et al Lung volume reduction surgery in selected patients with emphysema and pulmonary hypertension. Eur J Cardio-Thorac Surg. 2018;54:565-571. https://academic.oup.com/ejcts/article/54/3/565/4930722 10.1093/ejcts/ezy092 29538689

[8] Kaiwa Y , Kurokawa Y. [Lung volume reduction surgery for chronic pulmonary emphysema in elderly patients]. Kyobu Geka. 2005;58:709-713. 16097623

[9] Caviezel C , Schaffter N, Schneiter D, et al Outcome after lung volume reduction surgery in patients with severely impaired diffusion capacity. Ann Thorac Surg. 2018;105:379-385. 10.1016/j.athoracsur.2017.09.006 29223424

[10] Caviezel C , Schneiter D, Opitz I, Weder W. Lung volume reduction surgery beyond the NETT selection criteria. J Thorac Dis. 2018;10:S2748-S2753. 30210828 10.21037/jtd.2018.08.93 PMC6129809

[11] Vandervelde CM , Everaerts S, Weder W, et al Implementation of an enhanced recovery protocol for lung volume reduction surgery: an observational cohort study. Eur J Cardio-Thorac Surg. 2024;65:ezae109. https://academic.oup.com/ejcts/article/doi/10.1093/ejcts/ezae109/7632737 10.1093/ejcts/ezae109 38507704

[12] Weder W , Thurnheer R, Stammberger U, Bürge M, Russi EW, Bloch KE. Radiologic emphysema morphology is associated with outcome after surgical lung volume reduction. Ann Thorac Surg. 1997;64:313-320. 9262567 10.1016/S0003-4975(97)00564-X

[13] Vandervelde CM , Everaerts S, Weder W, et al Widening the selection criteria for lung volume reduction surgery. Eur Respir J. 2025;65:2400829. 39715645 10.1183/13993003.00829-2024 PMC11736308

[14] Seely AJE , Ivanovic J, Threader J, et al Systematic classification of morbidity and mortality after thoracic surgery. Ann Thorac Surg. 2010;90:936-942; discussion 942. https://linkinghub.elsevier.com/retrieve/pii/S0003497510010611 20732521 10.1016/j.athoracsur.2010.05.014

[15] Weder W , Tutic M, Lardinois D, et al Persistent benefit from lung volume reduction surgery in patients with homogeneous emphysema. Ann Thorac Surg. 2009;87:229-237. https://linkinghub.elsevier.com/retrieve/pii/S0003497508022236 19101303 10.1016/j.athoracsur.2008.10.012

[16] Thuppal S , Crabtree T, Markwell S, et al Pulmonary hypertension: a contraindication for lung volume reduction surgery? Ann Thorac Surg. 2020;109:902-906. https://linkinghub.elsevier.com/retrieve/pii/S0003497519315784 31610165 10.1016/j.athoracsur.2019.09.023

[17] Williams T , Gulack BC, Kim S, Fernandez FG, Ferguson MK. Operative risk for major lung resection increases at extremes of body mass index. Ann Thorac Surg. 2017;103:296-302. 27476820 10.1016/j.athoracsur.2016.05.057 PMC5182152

[18] DeCamp MM , Blackstone EH, Naunheim KS, et al; NETT Research Group. Patient and surgical factors influencing air leak after lung volume reduction surgery: lessons learned from the national Emphysema Treatment Trial. Ann Thorac Surg. 2006;82:197-207. https://linkinghub.elsevier.com/retrieve/pii/S0003497506003924 16798215 10.1016/j.athoracsur.2006.02.050

