# Supplementary material for: Expanding Lung Volume Reduction Surgery Indications: Outcomes in Patients Beyond Conventional National Emphysema Treatment Trial Criteria
Source: Interdiscip Cardiovasc Thorac Surg. 2025 Nov 18;40(12):ivaf274. doi: 10.1093/icvts/ivaf274 (PMC12782724; doi:10.1093/icvts/ivaf274)
Supplement: ivaf274_Supplementary_Data [file ivaf274_supplementary_data.docx]

**SUPPLEMENTARY MATERIAL**

**“EXPANDING LUNG VOLUME REDUCTION SURGERY INDICATIONS: OUTCOMES IN PATIENTS BEYOND CONVENTIONAL NETT CRITERIA”**

Christelle M. Vandervelde^1,2^, Anthony Meyers^1^, Anaïs David^1^, Sofian Bouneb^3,4^, Stephanie Everaerts^2,5^, Wim Janssens^2,5^, Walter Weder^6^, Laurens J. Ceulemans^1,2^

1. Department of Thoracic Surgery, University Hospitals Leuven, Leuven, Belgium
2. Department of Chronic Diseases and Metabolism Laboratory of Pneumology and Thoracic Surgery (BREATHE) KU Leuven, Leuven, Belgium
3. Department of Anaesthesiology University Hospitals Leuven, Leuven, Belgium
4. Department of Cardiovascular Sciences, division of Anaesthesiology and Algology, KU Leuven, Leuven, Belgium
5. Department of Respiratory diseases, University Hospitals Leuven, Leuven, Belgium
6. Department of Thoracic Surgery, Klinik Bethanien, Zurich, Switzerland

**Table S1: Beyond criteria patients**

|  | Patient characteristics | | | Cardiorespiratory functionality | | | | Morphology | | |
| --- | --- | --- | --- | --- | --- | --- | --- | --- | --- | --- |
| N° | Age ≥75 | BMI<18.5 | Thoracic interventions | FEV1 <20% | DLCO <20% | 6MWD <140m | sPAP >35 mmHg | | Homogeneous  Morphology |  |
| 1 | 58 | 20.86 | **Radiotherapy**  **(LUL)** | **19** | 34 | 505 | TTE: NA  RHC (sPAP): NA  RHC (mPAP): NA | | **homogeneous** |  |
| 2 | **75** | **17.37** | / | 25 | 33 | 290 | **TTE (sPAP): 49**  **RHC (sPAP): 29**  **RHC (mPAP): NA** | | Intermediate heterogeneous |  |
| 3 | 58 | **17.79** | **Pneumothorax**  **(chest tube)** | 39 | 29 | 435 | TTE (sPAP): 35  RHC (sPAP): NA  RHC (mPAP): NA | | Heterogeneous |  |
| 4 | 62 | **17.75** | / | **18** | 30 | 312 | TTE (sPAP): 18  RHC (sPAP): NA  RHC (mPAP): NA | | Heterogeneous |  |
| 5 | **77** | **16.51** | / | 33 | 23 | 332 | TTE (sPAP): 29  RHC (sPAP): NA  RHC (mPAP): NA | | Heterogeneous |  |
| 6 | 54 | **17.43** | / | 20 | 43 | 361 | TTE (sPAP): 35  RHC (sPAP): NA  RHC (mPAP): NA | | **Homogeneous** |  |
| 7 | 72 | 23.15 | / | **16** | 34 | 250 | **TTE (sPAP): 60**  **RHC (sPAP): 42**  **RHC (mPAP): NA** | | Intermediate heterogeneous |  |
| 8 | 65 | 28.41 | / | 24 | NA | **120** | TTE (sPAP): NA  RHC (sPAP): NA  RHC (mPAP): NA | | **Homogeneous** |  |
| 9 | 71 | **16.61** | / | 50 | 32 | 577 | **TTE (sPAP): 38**  **RHC (sPAP): NA**  **RHC (mPAP): NA** | | Heterogeneous |  |
| 10 | 56 | **13.89** | **Pleurodesis (bilat)**, **bullectomy (R), wedge resection (L)** | **13** | 28 | 318 | **TTE (sPAP): 36**  **RHC (sPAP): NA**  **RHC (mPAP): NA** | | Intermediate heterogeneous |  |
| 11 | **76** | 29.21 | / | 42 | 45 | 234 | **TTE (sPAP): 38**  **RHC (sPAP): NA**  **RHC (mPAP): NA** | | Intermediate heterogeneous |  |
| 12 | 56 | 25.90 | **Pneumothorax**  **(chest tube)** | 58 | 33 | 464 | **TTE (sPAP): 38**  **RHC (sPAP): 39**  **RHC (mPAP): 27** | | Heterogeneous |  |
| 13 | 73 | 19.81 | **Pneumothorax**  **(chest tube)** | 34 | 20 | 251 | **TTE (sPAP): 40**  **RHC (sPAP): NA**  **RHC (mPAP): NA** | | Heterogeneous |  |
| 14 | 72 | **15.89** | / | 34 | 25 | 290 | **TTE (sPAP): 39**  **RHC (sPAP): 37**  **RHC (mPAP): 24** | | Intermediate heterogeneous |  |
| 15 | 65 | **15.43** | / | 33 | **18** | 438 | **TTE (sPAP): 44**  **RHC (sPAP): 34**  **RHC (mPAP): 27** | | Intermediate heterogeneous |  |
| 16 | 64 | 19.82 | **Radiotherapy**  **(RUL)** | 27 | NA | 410 | **TTE (sPAP): 61**  **RHC (sPAP): NA**  **RHC (mPAP): NA** | | Intermediate heterogeneous |  |
| 17 | 74 | **18.27** | / | 54 | 29 | 433 | **TTE (sPAP): 53**  **RHC (sPAP): 40**  **RHC (mPAP): 27** | | Intermediate heterogeneous |  |
| 18 | 50 | 21.51 | **Hybrid ablation for atrial fibrillation (VATS, right)** | **16** | 22 | NA | **TTE (sPAP): 48**  **RHC (sPAP): 45**  **RHC (mPAP): 30** | | Intermediate heterogeneous |  |

**Legend:** Overview of individual patients beyond criteria, including age, body mass index (BMI), and presence of risk factors such as forced expiratory volume in 1 second (FEV₁) <20% predicted, diffusing capacity of the lung for carbon monoxide (DLCO) <20% predicted, and 6-minute walk distance (6MWD) <140 meters. Morphological classification is listed as homogeneous, heterogeneous, or intermediate heterogeneous. Pulmonary hypertension was assessed using systolic pulmonary artery pressure (sPAP) >35 mmHg. Right ventricular catheterization (RHC cathe), transthoracic echocardiography (TTE).

**Table S2: Surgical characteristics**

| **Operation Type** | Standard criteria n=227 | Beyond criteria n=21 | p-value |
| --- | --- | --- | --- |
| Unilateral, n (%) | 71 (31) | 9 (43) | 0.330 |
| One-staged bilateral, n (%) | 82 (36) | 6 (29) | 0.635 |
| Staged bilateral, n (%) | 37 (16) | 3 (14) | >0.999 |
| **Variabels** |  |  |  |
| Adhesions left, n (%) | 46 (39) | 3 (43) | >0.999 |
| Adhesions right, n (%) | 81 (51) | 6 (38) | 0.433 |
| No. of staplers left | 7.0 (3.0-19.0) n=116 | 8.0 (5.0-12.0) n=9 | 0.696 |
| No. of staplers right | 9.0 (4.0-30.0) n=159 | 8.0 (4.0-13.0) n=15 | 0.136 |
| Total pleurodesis, n (%) | 17 (7) | 3 (14) | 0.233 |
| Duration surgery (min)(total) | 89.0 (34.0-260.0) n=215 | 69.0 (43.0-151.0) n=19 | 0.112 |
| Duration surgery left lung (min) | 53.5 (30.0-162.0) n=118 | 48.0 (39.0-57.0) n=7 | 0.264 |
| Duration surgery right lung (min) | 59.0 (9.0-260.0) n=161 | 61.0 (29.0-86.0) n=16 | 0.505 |

**Legend:** Surgical characteristics are compared between standard- *versus* beyond-criteria procedures. Data are presented as median (range) or number (%). Min refers to minutes.

**Table S3: Functional outcomes**

| **Patients** | | **Standard criteria n=191** | **Beyond criteria n=18** | p-value |
| --- | --- | --- | --- | --- |
| **Functional characteristics** | |  |  |  |
| FEV_1_ (%pred) baseline | 31.0 (18.0-66.0) n=191 | 30.0 (13.0-58.0) n=18 | 0.308 |  |
| FEV_1_ (%pred) 3 months | 42.0 (16.0-100.0) n=175 | 40.0 (18.0-80.0) n=17 | 0.480 |  |
| FEV_1_ (%pred) 6 months | 39.0 (15.0-94.0) n=158 | 37.0 (21.0-82.0) n=15 | 0.639 |  |
| FEV_1_ (%pred) 12 months | 37.0 (15-109.0) n=112 | 42.0 (18.0-77.0) n=11 | 0.504 |  |
| RV (%pred) baseline | 222.5 (90.0-401.0) n=190 | 231.0 (178.0-392.0) n=18 | 0.626 |  |
| RV (%pred) 3 months | 166.0 (60.0-470.0) n=165 | 159.3 (51.0-273.0) n=15 | 0.952 |  |
| RV (%pred) 6 months | 178.0 (79.0-502.0) n=151 | 184.0 (77.0-303.0) n=13 | 0.797 |  |
| RV (%pred) 12 months | 172.0 (87.0-389.0) n=107 | 170.0 (65.0-316.0) n=10 | 0.753 |  |
| DLCO (%pred) baseline | 38.0 (20.0-71.0) n=185 | 29.5 (18.0-45.0) n=16 | **0.001** |  |
| DLCO (%pred) 3 months | 40.0 (19-95.0) n=168 | 29.9 (23.0-62.0) n=14 | **0.013** |  |
| DLCO (%pred) 6 months | 41.0 (16.0-74.0) n=146 | 30.0 (22.0-73.0) n=13 | **0.005** |  |
| DLCO (%pred) 12 months | 41.0 (15.0-96.0) n=105 | 33.0 (24.0-63.0) n=9 | 0.396 |  |
| 6MWD (m) baseline | 367.5 (106.0-608.0) n=180 | 332.0 (120.0-577.0) n=17 | 0.655 |  |
| 6MWD (m) 3 months | 424.0 (123.0-671.0) n=168 | 427.0 (180.0-629.0) n=13 | 0.806 |  |
| 6MWD (m) 6 months | 427.5 (47.0-693.0) n=146 | 409.0 (303.0-626.0) n=13 | 0.649 |  |
| 6MWD (m) 12 months | 415.0 (159.0-740.0) n=97 | 429.5 (211.0-627.0) n=8 | 0.751 |  |
| **Quality of life** | |  |  |  |
| CATscore baseline | 22.0 (6.0-39.0) n=148 | 20.0 (12.0-29.0) n=15 | 0.477 |  |
| CATscore 3 months | 14.0 (0.0-29.0) n=142 | 8.5 (4.0-27.0) n=10 | 0.132 |  |
| CATscore 6 months | 16.0 (0.0-36.0) n=131 | 13.0 (4.0-18.0) n=10 | 0.152 |  |
| CATscore 12 months | 16.0 (1.0-36.0) n=93 | 10.0 (6.0-21.0) n=7 | 0.086 |  |
| mMRC baseline | 3.0 (0.0-4.0) n=172 | 3.0 (2.0-4.0) n=16 | 0.178 |  |
| mMRC 3 months | 1.0 (0.0-4.0) n=150 | 1.0 (0.0-4.0) n=12 | 0.724 |  |
| mMRC 6 months | 1.0 (0.0-4.0) n=130 | 2.0 (0.0-3.0) n=12 | 0.963 |  |
| mMRC 12 months | 2.0 (0.0-4.0) n=106 | 0.0 (0.0-4.0) n=9 | 0.169 |  |
| SGRQ baseline | 62.0 (26.8-90.0) n=130 | 55.5 (28.0-76.0) n=11 | 0.517 |  |
| SGRQ 3 months | 41.0 (1.4-97.4) n=133 | 40.4 (22.0-72.8) n=8 | 0.676 |  |
| SGRQ 6 months | 48.0 (5.3-87.8) n=112 | 39.2 (24.0-59.6) n=7 | 0.508 |  |
| SGRQ 12 months | 46.0 (1.0-84.3) n=87 | 25.0 (1.0-45.0) n=7 | **0.007** |  |

**Legend:** Functional outcomes and quality of life scores over time are presented for patients: standard *versus* beyond criteria. Data are shown as median (range) or number (%). Categorical data: Fisher’s exact test. Continuous variables: Mann-Whitney tests. 6MWD: 6-Minute Walk Distance, CAT: COPD Assessment Test, DLCO: Diffusing Capacity of the Lung for Carbon Monoxide, FEV₁: Forced Expiratory Volume in 1 Second, mMRC: Modified Medical Research Council Dyspnea Scale, RV: Residual Volume, and SGRQ: St-George’s Respiratory Questionnaire.

**Table S4: Beyond criteria baseline *versus* 12 months functional outcome**

| **Beyond criteria (n=18)** | **Baseline** | **12 months** | **p-value** |
| --- | --- | --- | --- |
| FEV_1_ (%pred) | 30.0 (13.0-58.0) n=18 | 42.0 (18.0-77.0) n=11 | **0.038** |
| RV (%pred) | 231.0 (178.0-392.0) n=18 | 170.0 (65.0-316.0) n=10 | 0.097 |
| DLCO (%pred) | 29.5 (18.0-45.0) n=16 | 33.0 (24.0-63.0) n=9 | 0.081 |
| 6MWD (m) | 332.0 (120.0-577.0) n=17 | 429.5 (211.0-627.0) n=8 | 0.244 |
| mMRC (score) | 3.0 (2.0-4.0) n=16 | 0.0 (0.0-4.0) n=9 | **0.002** |
| CAT (score) | 20.0 (12.0-29.0) n=15 | 10.0 (6.0-21.0) n=7 | **0.003** |
| SGRQ (points) | 55.5 (28.0-76.0) n=11 | 25.0 (1.0-45.0) n=7 | **0.001** |

**Legend:** Continuous variables: Mann-Whitney tests.

**Table S5: Standard criteria baseline *vs* 12 months functional outcome**

| **Standard criteria (n=191)** | **Baseline** | **12 months** | **p-value** |
| --- | --- | --- | --- |
| FEV_1_ (%pred) | 31.0 (18.0-66.0) n=191 | 37.0 (48-109.0) n=112 | **<0.001** |
| RV (%pred) | 222.5 (90.0-401.0) n=190 | 172.0 (87.0-389.0) n=107 | **<0.001** |
| DLCO (%pred) | 38.0 (20.0-71.0) n=185 | 172.0 (87.0-389.0) n=107 | **0.027** |
| 6MWD (m) | 367.5 (106.0-608.0) n=180 | 415.0 (159.0-740.0) n=97 | **0.002** |
| mMRC (score) | 3.0 (0.0-4.0) n=172 | 2.0 (0.0-4.0) n=106 | **<0.001** |
| CAT (score) | 22.0 (6.0-39.0) n=148 | 16.0 (1.0-36.0) n=93 | **<0.001** |
| SGRQ (points) | 62.0 (26.8-90.0) n=130 | 46.0 (1.0-84.3) n=87 | **<0.001** |

**Legend:** Continuous variables: Mann-Whitney tests.**Table S6: Missing follow-up data**

|  | **3 months** | | **6 months** | | **12 months** | |
| --- | --- | --- | --- | --- | --- | --- |
| Missing follow-up data | Standard criteria | Beyond criteria | Standard criteria | Beyond criteria | Standard criteria | Beyond criteria |
| No visit yet | 0 | 0 | 11 | 1 | 59 | 4 |
| Acute exacerbation | 4 | 0 | 3 | 1 | 0 | 0 |
| No follow-up in our center | 4 | 0 | 5 | 0 | 1 | 0 |
| No show | 2 | 1 | 8 | 1 | 14 | 1 |
| Death | 3^A^ | 0 | 5 | 0 | 10 | 1 |
| Other | 2 | 0 | 1 | 0 | 0 | 0 |
| **Total** | **15** | **1** | **33** | **3** | **84** | **6** |

**Legend:** Overview of patients lost to follow-up at 3, 6, and 12 months after lung volume reduction surgery (LVRS), comparing those treated within standard criteria versus beyond criteria. Main reason for missing data was no planned follow-up visit yet, reasons for loss to follow-up were acute exacerbation, death, follow-up in another center, no-show, or other causes. (A) Causes of death included acute on chronic kidney injury and two cases of COVID-19.

**Figure S1: Percentage of beyond criteria procedures over time**

**
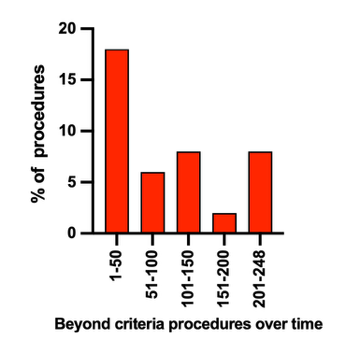
**
